# Low Dose Pregabalin Improves Gastrointestinal Symptoms of Crohn's Disease

**DOI:** 10.1155/2024/3744500

**Published:** 2024-03-15

**Authors:** Antonio Maria D'Onofrio, Federica Di Vincenzo, Gaspare Filippo Ferrajoli, Franco Scaldaferri, Giovanni Camardese

**Affiliations:** ^1^Department of Neuroscience, Section of Psychiatry, Università Cattolica del Sacro Cuore, Rome 00168, Italy; ^2^UOS Malattie Infiammatorie Croniche Intestinali, CEMAD Digestive Diseases Center, Fondazione Policlinico Universitario “A. Gemelli” IRCCS, Università Cattolica del Sacro Cuore, Rome 00168, Italy; ^3^Dipartimento di Medicina e Chirurgia Traslazionale, Università Cattolica del Sacro Cuore, Rome 00168, Italy

## Abstract

Inflammatory bowel diseases (IBD), including Crohn's disease and ulcerative colitis, are lifelong conditions with no definite cure. Several studies demonstrated that patients with IBD more frequently experience symptoms of common mental disorders, such as anxiety and depression, because of bidirectional communication through the gut-brain axis and the chronicity of symptoms, as well as because of impaired quality of life and reduced social functioning. However, psychological conditions of affected patients are often underestimated and not fully considered. Herein, we present the case of a 37-year-old woman with Crohn's disease and a mild depressive condition, characterized by anxious distress, tachycardia, tachypnea, tremors, sweating, avoidant behaviors, and intestinal somatizations (diarrhea), who was treated with Pregabalin upon indication of the referring psychiatrist. Following the beginning of the treatment, the patient rapidly reported an improvement in the overall clinical symptoms as well as a better management of psychic and physical anxiety with a marked reduction in diarrheal discharges under stress at work. After 6 months of Pregabalin therapy, we additionally observed an improvement in Crohn's disease activity, both clinically, in the laboratory, and endoscopically. Our case showed that patients with Crohn's disease and anxiety problems may benefit from low-dose Pregabalin medication to improve both their mental and physical condition.

## 1. Background

Inflammatory bowel diseases (IBD), including Crohn's disease (CD) and ulcerative colitis (UC), are chronic, relapsing immune-mediated diseases with a varying and sometimes severe disease course. IBD is often diagnosed in early adulthood and can lead to a substantial decline in the quality of life of affected patients. It has been suggested that patients with IBD are more likely to experience symptoms of common mental disorders such as anxiety and depression due to bidirectional communication via the gut-brain axis, the chronicity of symptoms, and because of impaired quality of life and reduced social functioning [1–3]. A recent meta-analysis showed that among IBD patients, the prevalence of anxiety symptoms was 32.1% and of depression was 25.2%. Interestingly, patients with Crohn's disease had higher odds of anxiety and depression symptoms than patients with ulcerative colitis [4]. Despite the increased depression and anxiety in IBD patients, most of them do not receive the mental health treatment they may require [5, 6]. Several studies demonstrated that anxiety and depression further reduce the quality of life of IBD patients, and the course of disease is worse in patients with depression [6–8]. Therefore, physicians should be more aware of the psychological symptoms of their patients and provide them with appropriate psychological and pharmacological support when needed.

The primary aim of this study is to explore, using a clinical case that has captured our attention, how addressing the mental health of patients with IBD using a safe medication, such as Pregabalin, could improve their gastrointestinal (GI) symptoms through the well-documented and increasingly studied gut-brain axis.

## 2. Case Presentation

Herein, we present the case of a 37-year-old white woman diagnosed with Crohn's disease (Montreal classification A2B1L3p) in 2006 who was referred to our psychiatric clinic by her trusty gastroenterologist.

The patient presented at our clinic in April 2021. During the interview with the referring psychiatrist, the patient dated the beginning of her psychopathological symptoms back to 2006 (in combination with her Crohn's disease diagnosis), when she started to experience symptoms of anxiety, such as worries, tachycardia, tachypnea, tremors, sweating, avoidant behaviors, as well as intestinal somatizations (diarrhea). At that time, she had been treated with an unspecified combination of anxiolytic and antidepressant medication for her symptoms, from which she had not found any improvement. Thus, she decided to discontinue the medications on her own. The patient also described a continuous stressful period of her life that had lasted since her father's death and had taken on the characteristics of unprocessed grieving. Furthermore, since 2006, she has made the decision to devote herself exclusively to work, taking on considerable responsibilities. The patient's experiences revealed an essential inclination to control and a fine perfectionism: when things did not meet her standards, she tended to show nervous episodes with predominantly somatized anxiety and exacerbation of GI symptoms.

Her gastroenterological history started one year before the diagnosis of Crohn's disease. The patient was initially treated with corticosteroids, followed by azathioprine until 2012. Due to a flare-up of the disease in 2012, the patient was switched to Infliximab, which she continued with clinical benefit for 6 years. In 2014, she developed an uncomplicated perianal fistula that was successfully managed with surgical curettage and the placement of a loose seton. Due to the development of a secondary non-response to Infliximab and de-novo enteroarthritis of the small joints of her hands, she was switched to Ustekinumab for few months. Nevertheless, she never achieved a clinical response to Ustekinumab, and she developed erythema nodosum. Therefore, Adalimumab therapy was started in 2019. Due to the persistence of gastrointestinal symptoms, Vedolizumab was added off-label to Adalimumab in September 2020 for compassionate use, and both are currently ongoing.

Her previous clinical history was otherwise unremarkable.

Her family history was characterized by psychopathological disorders in some family members, including an anxious-depressive syndrome in her mother, an anxiety disorder in her brother, and an alcohol use disorder in her father, who eventually developed liver cirrhosis and died of liver cancer.

At the medical evaluation, the patient presented a slightly depressed mood and a moderate psychological and somatic anxiety. Neither changes in thought form nor content were detected, nor direct or indirect indicators of any abnormal perceptions. She rejected any thoughts, planning or intent of self-harm/suicide. Her insight appeared to be adequate. On the psychometric scales that investigate anxious and depressive symptoms, the patient presented the following scores: Hamilton Anxiety Rating Scale (HAM-A) equal to 23 (this score indicates mild to moderate anxiety) [9] and Hamilton Depression Rating Scale (HAM-D) equal to 11 (this score indicates a mild depression) [10]. As a result, a mild depressive disorder with anxious distress was diagnosed as a Cluster B personality disorder. The patients also reported ongoing GI symptoms, including diarrhea, with 5-6 bowel movements per day, mild abdominal pain, and a low energy level.

Given her tendency for control and a lack of compliance with any psychopharmacological treatment, an anxiolytic therapy based on Pregabalin up to 75 mg/day and S-adenosyl-methionine was proposed, with the goal of avoiding the potential GI side effects associated with selective serotonin reuptake inhibitors (SSRI) therapy. A psychoeducational interview was also conducted in order to motivate the patient to begin a psychotherapy course.

She accepted the recommended treatment plan and started the psychopharmacological therapy in May 2021, as well as the psychotherapy, attending all check-up visits. She quickly reported an amelioration of her general wellbeing and a better management of her psychic and physical anxiety with a clear reduction in diarrheal discharges under stress at work.

Notably, with the improvement of the psychopathological symptomatology, the patient also experienced a progressive clinical, laboratory, and endoscopic improvement of Crohn's disease as well. In May 2021, the patient presented with a Harvey-Bradshaw Index (HBI) of 9, driven by diarrhea and mild abdominal pain, a C-reactive protein (CRP) of 21 mg/L, and a SES-CD of 17 at the last colonoscopy, performed a few months before. After two months of pregabalin therapy, CRP normalized and HBI dropped to 4, with a significant reduction in the number of daily bowel movements, as well as abdominal pain. At the colonoscopy performed 6 months afterwards, a significant improvement in colonic inflammation was observed, with a SES-CD of 6. Therefore, the clinical, laboratory, and endoscopic improvement of Crohn's disease could be attributed more to the psychopathological treatment than to the combination biological therapy with Adalimumab and Vedolizumab that the patient had been following, respectively, for 2-years and 8 months when Pregabalin was started.

## 3. Discussion

In the current study, we reported the case of a patient who, after the introduction of Pregabalin therapy, experienced an improvement not only in her anxiety, mood, and quality of life but also in her gastrointestinal symptoms, as well as laboratory and endoscopic findings of inflammation related to her Crohn's disease. Indeed, two months after the beginning of Pregabalin, normalization of CRP was observed, while at six months there was endoscopic improvement of the disease together with the symptomatologic improvement reported by the patient. In addition to Pregabalin therapy, the patient was also receiving Adalimumab and Vedolizumab at stable doses for 2 years and 8 months since the start of psychopathological treatment, respectively, without experiencing similar improvement in GI symptoms.

Inflammatory bowel disease is notoriously associated with increased rates of anxiety disorders (20.5% of IBD patients) and general anxiety symptoms (35.1% of affected subjects). Furthermore, patients with active disease experience a 75.6% higher prevalence of these symptoms than patients with disease in remission [4]. Previous studies suggested that daily stress could be a risk factor for diseases such as IBD and that the gut-brain axis could play a central role in their development. Notably, stress seemed to increase intestinal permeability, visceral sensitivity, alter GI-motility and lead to mast cell activation with the release of proinflammatory mediators [11]. Therefore, addressing psychopathological comorbidities in patients with IBD is crucial for the control of gastrointestinal symptoms.

Pregabalin is a structural analog of the inhibitory neurotransmitter *γ*-aminobutyric acid (GABA). It binds to the alpha2delta subunit of presynaptic voltage-gated calcium channels, modulating the release of excitatory neurotransmitters [12]. It is widely used in the treatment of epilepsy, neuropathic pain, fibromyalgia [13–17], and anxiety disorders (generalized anxiety disorder and social anxiety disorder). Previous studies demonstrated that Pregabalin has an anti-inflammatory effect, by influencing the production of proinflammatory cytokines, such as interleukin (IL)-2, IL-6, IL-8, IL-1*β*, and tumor necrosis factor (TNF)*α* [18] by limiting neutrophil recruitment [19] and reducing the damages after oxidative stress [20]. Indeed, through the modulation of substance P, Pregabalin has shown to inhibit nuclear factor kappa B (NF-*κ*B) and its target genes, such as cyclooxygenase-2 and p38 mitogen-activated protein kinase [21], thereby leading to decreased vascular permeability, leukocyte recruitment, and the release of proinflammatory cytokines [22].

Previous studies demonstrated that colonic inflammation determines the overexpression of NMDA receptors in the enteric nervous system [23]; by binding to these receptors, glutamate causes the accumulation of oxygen reactive species, the activation of NF-*κ*B, and the increase of pro-inflammatory cytokines, such as TNF-*α* and IL-1*β* [24]. NMDA receptor antagonist therapy normalized bowel movements and microcirculation in mice model of experimental colitis [25]. Notably, recent studies suggest that Pregabalin treatment can inhibit the release of glutamate and attenuate N-methyl-D-aspartate (NMDA) receptor-mediated synaptic transmission [26], thereby attenuating experimental colitis. Accordingly, an interesting study conducted by Motavallian Azadeh et al. in mice models of acetic acid-induced colitis showed that the administration of different doses of Pregabalin (30, 50, and 100 mg/kg; intraperitoneally, respectively) significantly decreased the severity of macroscopic and microscopic colonic damages, including the percentage of necrosis, ulcer severity, and extension and total colitis index, compared to the colitis control group. The authors also described a reduction of colonic concentration of tumor necrosis factor-alpha, interleukin-6, interleukin-1 beta, and myeloperoxidase activity after Pregabalin administration [27].

**Figure 1:**
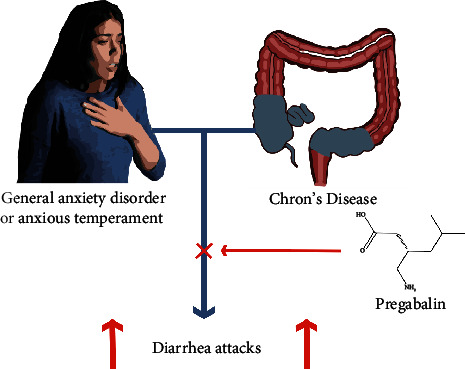
The effect of Pregabalin in a patient with anxious features and Chron's disease.

Therefore, pregabalin appears to be one of the most suitable drugs for the treatment of anxiety in a subset of patients with IBD (Figure 1), considering its pharmacodynamic and pharmacokinetic properties, its possible side effects, which do not commonly involve the gastrointestinal tract [17], as well as the absence of any need for special blood chemistry tests to be done during its intake and its ease of use even by physicians not specialized in psychiatry. Moreover, pregabalin does not induce abuse behavior and is unlikely to provide withdrawal.

A psychiatrist, clinical psychologist, or trained gastroenterologist should screen emotionally vulnerable IBD patients for the presence of an anxiety condition, an anxious temperament, or very stressful life circumstances. Tools for identifying individuals who may benefit from psychopharmacological anxiolytic therapy (together with appropriate psychotherapeutic therapies) are self-administered psychometric scales such as the Hospital Anxiety and Depression Scale (HADS) [28] which could be appropriate in a gastroenterology setting. The management of these scales, along with that of IBD must become more comprehensive, giving equal weight to physical and mental outcomes.

However, our study presents some limitations. The main limitations include the absence of a colonoscopy performed right before the beginning of pregabalin treatment, as well as the concomitant treatment of the patients with two biological drugs, which; however, did not yield the expected effect on the control of GI symptoms. Furthermore, due to the single-patient study design, it is not possible to establish causality between pregabalin intake and improvement in gastrointestinal symptoms. Unfortunately, before the initiation of Pregabalin therapy, no fecal samples were collected from the patient; thus, we could not study the effect of this drug on the modulation of the intestinal microbiota and, therefore, on the gut-brain axis. Further research is needed to deeply investigate the effect of Pregabalin on the gut microbiota and its modulation of the gut-brain axis in IBD patients.

In conclusion, further studies and clinical trials are required to assess the effects of this medication on gastrointestinal outcomes in a large cohort of IBD patients, either with an established diagnosis of anxiety disorders or even with an anxious temperament, in whom IBD could trigger an overt psychiatric disorder, thus initiating a vicious cycle where psychiatric and GI symptoms are self-maintained. Nonetheless, in the meantime, we suggest using this medication in a subset of IBD patients with symptoms of anxiety or depression as well as ongoing gastrointestinal disorders in order to benefit from its anti-inflammatory effects and to avoid the possible GI side effects of other classes of medications.

## Data Availability

The data that support the findings of this study are available from the corresponding author, [FDV], upon reasonable request.
